# Crosstalk between m6A mRNAs and m6A circRNAs and the time-specific biogenesis of m6A circRNAs after OGD/R in primary neurons

**DOI:** 10.1080/15592294.2023.2181575

**Published:** 2023-03-01

**Authors:** Chi Zhang, Huan Jian, Shenghui Shang, Lu Lu, Yongfu Lou, Yi Kang, Hong Bai, Zheng Fu, Yigang Lv, Xiaohong Kong, Xueying Li, Shiqing Feng, Hengxing Zhou

**Affiliations:** a Department of Orthopaedics, Qilu Hospital, Shandong University Centre for Orthopaedics, Advanced Medical Research Institute, Cheeloo College of Medicine, Shandong University, Jinan, China; Shandong University Centre for Orthopaedics, Advanced Medical Research Institute, Cheeloo College of Medicine, Shandong University, Jinan, China; b Department of Orthopaedics, Tianjin Medical University General Hospital, International Science and Technology Cooperation Base of Spinal Cord Injury, Tianjin Key Laboratory of Spine and Spinal Cord, Tianjin, China; c Key Laboratory of Immuno-Microenvironment and Disease of the Educational Ministry of China, Department of Immunology, Tianjin Medical University, Tianjin, China

**Keywords:** Epitranscriptomics, N6-methyladenosine, neuron, OGD/R, circRNA

## Abstract

Cerebral ischaemiareperfusion injury is an important pathological process in nervous system diseases during which neurons undergo oxygenglucose deprivation and reoxygenation (OGD/R) injury. No study has used epitranscriptomics to explore the characteristics and mechanism of injury. N6methyladenosine (m6A) is the most abundant epitranscriptomic RNA modification. However, little is known about m6A modifications in neurons, especially during OGD/R. m6A RNA immunoprecipitation sequencing (MeRIPseq) and RNA-sequencing data for normal and OGD/R-treated neurons were analysed by bioinformatics. MeRIP quantitative real-time polymerase chain reaction was used to determine the m6A modification levels on specific RNAs. We report the m6A modification profiles of the mRNA and circRNA transcriptomes of normal and OGD/R-treated neurons. Expression analysis revealed that the m6A levels did not affect m6A mRNA or m6A circRNA expression. We found crosstalk between m6A mRNAs and m6A circRNAs and identified three patterns of m6A circRNA production in neurons; thus, distinct OGD/R treatments induced the same genes to generate different m6A circRNAs. Additionally, m6A circRNA biogenesis during distinct OGD/R processes was found to be time specific. These results expand our understanding of m6A modifications in normal and OGD/R-treated neurons, providing a reference to explore epigenetic mechanisms and potential treatments for OGD/R-related diseases.

## Introduction

Cerebral ischaemia-reperfusion injury (IRI) is the main pathological process in many central nervous system disorders, such as traumatic brain injury (TBI) [1], acute ischaemic stroke [2] and spinal cord injury [3]. Ischaemia and blood flow reperfusion cause damage at the ischaemic site. Neurons undergo oxygen-glucose deprivation/reoxygenation (OGD/R), which results in material and energy metabolism disorders, subsequently affecting neuronal biological processes such as survival [4], apoptosis [5], and autophagy [6]. Regarding OGD/R, the dysfunction of biological processes leads to the occurrence and aggravation of diseases [4–6]. Therefore, it is of great significance to study the changes in neurons undergoing the OGD/R process.

N6-methyladenosine (m6A) is the most prevalent post-transcriptional modification in messenger RNA (mRNA) [7–9] and non-coding RNAs [10], and m6A modification on mRNAs regulates various phenotypes of cells. For example, m6A regulates durable neoantigen-specific immunity [11], spermatogonial differentiation and meiosis initiation [12], and symmetric commitment and may affect the fate of haematopoietic stem cell (HSC) differentiation [13]. m6A also plays a vital role in neurons, as the m6A modification of axonal mRNAs can control local mRNA translation [14]. In the context of neurological damage, m6A modification on mRNA is required for axon regeneration and injury-induced protein synthesis, which provide a way to repair injury [15]. YTHDF1 facilitates the translation of m6A-methylated neuronal mRNAs in response to neuronal stimulation, contributing to learning and memory [16]. These findings show that m6A modification on mRNA affects some functions in neuron. However, the regulation of mRNA m6A modification and the changes of its functions under OGD/R were largely unknown. Therefore, in-depth exploration of the characteristics of m6A modification on mRNA in neurons under different circumstances is highly significant for understanding the physiological and pathological processes of the nervous system and potential treatments for neurological diseases.

Circular RNAs (circRNAs) are a class of closed-ring non-coding RNAs [17,18], most of which are derived from known protein-coding genes [19]. circRNAs are tissue and cell-specific and act as important pathophysiological regulators of the nervous system [20]. circRNAs are abundant in neurons and mostly related to the formation and regulation of synapses during brain development [20,21]. For example, circHECTD1 functions as a sponge of miR-142, resulting in the inhibition of TIPARP expression with the subsequent inhibition of astrocyte activation via autophagy in transient middle cerebral artery occlusion (tMCAO) models [22]. Additionally, circRar1 induces the upregulation of caspase8 and p38 (apoptosis-associated factors) via modulation of the target microRNA (miRNA) miR-671 [23]. Although many roles of circRNAs in neurons are recognized, we still know little with regard to the characteristics and functions of circRNAs in neurons under different OGD/R conditions.

Previous studies have revealed that circRNA can acquire m6A modifications [24–28]. m6A promotes circRNA production, and the resulting m6A circRNAs may compensate for the massive degradation of mRNAs in late spermatogenesis [28]. m6A modification of circRNAs can drive translational initiation [24]. m6A circRNAs are endoribonucleolytically cleaved by the YTHDF2-HRSP12-RNase P/MRP axis in a novel molecular mechanism of multilayered gene regulation mediated by m6A modification [27]. In addition to the important role of m6A-modified circRNAs in physiological processes, recent studies have also reported the existence of regulatory functions of m6A circRNAs in a variety of diseases. m6A circNSUN2 regulates cytoplasmic export and stabilizes HMGA2 to promote colorectal carcinoma (CRC) liver metastasis (LM) [26]; In colorectal cancer, another METTL3-induced circ1662 promoted CRC cell invasion and migration by accelerating YAP1 nuclear transport [29]. In gastric cancer, m6A modification of circORC5 repressed growth and invasion of GC cells progression by regulating miR-30c-2-3p/AKT1S1 axis [30]. Notably, m6A modification of circRNA has cell-type-specific roles during the normal physiological process and also plays regulatory roles in a variety of diseases. Some connections exist between m6A mRNA biogenesis and m6A circRNA biogenesis, Liu et.al found that m6A modification promoted the back-splicing of circIGF2BP3, paralleled by a decrease in its precursor transcript in A549 cells [31]. However, the circumstances of such preferential events in different cells and the mechanisms by which they occur remain unclear.

Here, we examined the transcriptome-wide modification of m6A mRNAs and m6A circRNAs in normal neurons and different OGD/R-treated neurons. Our results revealed no relationship between the m6A level and m6A RNA (m6A mRNA and m6A circRNA) expression. However, evidence of crosstalk between m6A mRNAs and m6A circRNAs was obtained in this study. We also discovered that the production of m6A circRNAs has time specificity.

## Materials and methods

### Animals

Primary cerebral neurons were harvested from C57BL6 mice purchased from the Laboratory Animal Centre, Academy of Military Medical Science (Beijing, China). All of the experiments were performed in adherence with the National Institutes of Health Guidelines on Laboratory Animals and were approved by the Medical Ethics Committee of Qilu Hospital of Shandong University.

### Primary cerebral neuron isolation and culture

Primary cultures of cerebral neurons were obtained as described previously [32]. Briefly, mouse foetuses (embryonic day [E] 17) were removed from the uterus, and the individual foetuses were freed from the embryonic sac. Brain and cortical tissues were dissected and placed in high-glucose Dulbecco’s modified Eagle’s medium without phenol red (DMEM-HG; Gibco, Grand Island, NY, USA; Cat. No. 31053028). Papain solution (10 U/mL; Sigma-Aldrich, St. Louis, MO, USA; Cat. No. LS003126) was added to these cerebral tissues, which were then incubated for 15 min at 37°C in a 5% CO_2_ incubator. Dissociated cortical cells were plated on poly-L-lysine (Sigma-Aldrich; Cat. No. P4832)-coated cell culture dishes and cultured in DMEM-HG (Gibco, Grand Island, NY, USA; Cat. No. 31053028) containing 10% foetal bovine serum (Gibco, Australia; Cat. No. 10099141) and 1% penicillin/streptomycin (P/S; Invitrogen, Carlsbad, CA, USA; Cat. No. 15140148) at a density of 1.0 × 10^6^ cells/mL. After 4 h of seeding, the medium was changed to neurobasal medium (NM; Gibco, Carlsbad; Cat. No. 21103049) supplemented with B-27 (Gibco, Grand Island, NY; USA Cat. No. 17504044). The cells were cultured in a humidified incubator at 37°C with 5% CO_2_. The medium was changed every 3 days. Cultures were used for *in vitro* experiments after 7 days.

### Immunofluorescence

After 6 days of culture, neurons were fixed with 4% paraformaldehyde for 10 minutes, and the cell membrane was permeabilized for 5 min in 0.1% Triton X-100. To reduce non-specific binding, 10% goat serum (Solarbio, Beijing, China, Cat. No. SL038) was added for 1 h at room temperature. Then, cells were incubated with primary antibodies, including β-III tubulin (1:400, Abcam, Cambridge, UK, Cat. No. ab78078), labels neurons and axons, GFAP (1:400, Abcam, Cambridge, UK, Cat. No. ab7260) labels astrocyte, incubated overnight at 4°C. The corresponding secondary antibody were incubated at room temperature for 1 h. After washing 3 times with PBS, the cells were incubated with DAPI (Beyotime, Shanghai, China, Cat. No. C1002) to label nucleus for 10 minutes, and images were obtained under a fluorescence microscope (Olympus, Tokyo, Japan). Under the microscope, the ratio of β-III tubulin-positive cells to DAPI cells was counted in three random fields to obtain the purity of the extracted neurons under three different perspectives.

### OGD/R Modelling

The OGD/R model was established using a previously described method with slight modifications [4,33,34]. Phosphate-buffered saline (PBS; Sigma-Aldrich; Cat. No. D8537) supplemented with 1% P/S was used to wash the cultured primary cerebral neurons twice after 7 days of culture. Dulbecco’s modified Eagle’s medium without glucose (Gibco, Grand Island, NY, USA; Cat. No. 31053028) was added to the dishes. Next, the neurons were subjected to the GENbag anaerobic incubation system (bioMérieux SA, France; Cat. No. 45534) at 37°C. The cultures were kept separately under hypoxic conditions for 1.5 and 3 h to achieve oxygen-glucose deprivation. Thereafter, the neurons were allowed to recover with normal serum-free medium (neurobasal medium) under normal incubation conditions (37°C, 5% CO_2_) for 24 h. Neurons cultured in normal serum-free medium under normoxic conditions served as controls.

### RNA Isolation

The total RNA was extracted from the primary cultured neurons using TRIzol reagent (Invitrogen, Carlsbad, CA, USA; Cat. No. 15596018) according to the manufacturer’s protocol. A NanoDrop ND-1000 system (Thermo Fisher Scientific, Waltham, MA, USA) was used to measure the RNA concentration in each sample. The OD260/OD280 value ratio was assessed as an index of the RNA purity, and OD260/OD280 values ranging from 1.8 to 2.1 indicated that the RNA met the qualifications for purity. The RNA integrity was measured using denaturing agarose gel electrophoresis.

### RNA dotblot

RNA samples were denatured at 95°C for 5 min, followed by chilling on ice immediatedly. Samples were spotted on the membrane (Amersham Hybond-N+, GE) and dried for 5 min at room temperature, then UV-crosslinked (Auto crosslink mode) for 1 h. After UV crosslinking, the membranes were washed by TBST for 3 times. Membranes were blocked in blocking buffer (5% non-fat milk in TBST)) for 1 h at room temperature, then incubated with anti-m6 A antibody (1:1000, Cell Signalling Technology, Danvers, MA, USA. Cat. No. 56593) at 4°C overnight. Membranes were washed with TBST 3 times and then incubated with HRP-linked secondary anti-rabbit IgG antibody (1: 2000, Cell Signalling Technology, Danvers, MA, USA. Cat. No. 7074S) for 1 h at room temperature. After 3 times washes with TBST, the membrane was visualized by using Immobilon Western Chemiluminescent HRP Substrate (Merck Millipore, Cat. No. WBKLSO500).

### Preparation of the m6A RNA Immunoprecipitation Sequencing (MeRIP-seq) Library

MeRIP-seq was performed by Cloudseq Biotech Inc. (Shanghai, China) according to a published procedure with slight modifications [35]. Briefly, three biological replicates were used for the control, OGD/R 1.5 h and OGD/R 3 h groups. Isolated RNA was chemically fragmented into fragments of approximately 100 nucleotides in length using fragmentation buffer (Illumina, Inc., CA, USA). A Magna MeRIP™ m6A Kit (Merck Millipore, MA, USA; Cat. No. 17–10499) was used to perform m6A RNA immunoprecipitation (IP) according to the manufacturer’s recommendations. IP buffer (Cat. No. CS220009), Magna ChIP Protein A/G Magnetic Beads (Cat. No. CS203152) and an anti-m6A antibody (Cat. No. MABE1006) were used to prepare the magnetic beads for IP. Next, the MeRIP reaction mixture was configured according to the manufacturer’s guidelines, and the fragmented RNA was included in this mixture. The magnetic beads and MeRIP reaction mixture were combined in tubes, and all of the tubes were incubated with rotation for 2 h at 4°C. Subsequently, elution buffer was prepared according to the manufacturer’s instructions and was used to elute bound RNA from the beads using the m6A antibody in IP buffer. Input samples without IP and m6A IP samples were both used for library construction using the NEBNext® Ultra™ II Directional RNA Library Prep Kit (New England Biolabs, Inc., MA, USA). Library sequencing was performed using an Illumina HiSeq 4000 instrument (Illumina, Inc., CA, USA) with 150-bp paired-end reads.

### MeRIP Quantitative Real-Time Polymerase Chain Reaction (qRT-PCR)

RNA subjected to MeRIP analysis as described above was harvested. One-step qRT-PCR for RNA was conducted according to the instructions for the analysis of MeRIP-analysed RNA in the Magna MeRIP™ m6A Kit. Briefly, 2 μL of the MeRIP-analysed RNA sample was added to 18 μL of a master reaction mixture (prepared according to the manufacturer’s instructions) in PCR plates. Optical tape was used to seal the plate, and qRT-PCR was performed. The qRT-PCR parameters were set according to the manufacturer’s instructions. The sequences of the primers used are shown in Table 1.

**Table 1.** Primer sequences for MeRIP-qPCR.

Gene	Primer name	Primer sequence
Ccdc93	1-Forward	CCCAGCAAACCAGAGATGTT
	1-Reverse	ATCCGACAAGGTGGTACAGC
Tmem38b	2-Forward	GCCCGATCTTGTCTGATCTC
	2-Reverse	AAGCCTACAGCACCCGGTAT
Wee1	3-Forward	AACGGGCACTCTTCACAGAT
	3-Reverse	GCTGACAGAGCGGTTCATTT
Dst	4-Forward	TAGAAGCTCAGGCTGCAACA
	4-Reverse	GATCAACGATGCCATGAAGA
Exoc8	5-Forward	CGTGCTACAGATGCGTCATT
	5-Reverse	GCATTTTTCAGAGGGCACTT
Myo18a	6-Forward	ATCCAGGGATGAAATTGTGG
	6-Reverse	CTCAGCTCGCTAAGCTCTGG
Scn2a1	7-Forward	TCTTTCAGCTCGGACAACCT
	7-Reverse	CAAAATCAATCCCCTTCTGC
Ranbp2	8-Forward	TCTGTGGGTGGAAATGATGA
	8-Reverse	GCTGACCCATCTTCAAAAGC
Dnajc5	9-Forward	ATGAAGTGCGTAGCATGCAG
	9-Reverse	AGCACGTCCACAACACAGAC
chr11:26387228-26470948+	10-Forward	GTACCCTGAGGGGTCGTTCT
	10-Reverse	GCTACTTCTCCAGGCTCTGC
chr17:29631671-29634768+	11-Forward	GAAAAGGACAAGGTGCAAGC
	11-Reverse	TCCGAGCAGATAATGCACTG
chr7:97705589-97718381+	12-Forward	CGGCTTTCCTGTTTCATTTT
	12-Reverse	GAAAGGGCCTAGGCACAAAT

### Gene Ontology (GO) and Kyoto Encyclopaedia of Genes and Genomes (KEGG) pathway analyses

GO and KEGG pathway analyses were performed using the Database for Annotation, Visualization and Integrated Discovery on differentially methylated m6A mRNAs and m6A circRNAs in the OGD/R 1.5 h and OGD/R 3 h groups [36,37]. GO enrichment was used to assess the enriched cellular component (CC), molecular function (MF) and biological process (BP) terms for each group. The enriched pathways in each group were determined by KEGG analysis. Both the GO and KEGG analyses were conducted using R software with the clusterProfiler package (v3.12.0) [38], and Fisher’s exact test was used to select the significant GO terms and pathways. A p-value < 0.05 was used as the threshold of significance for GO terms and enriched pathways.

### Plasmid construction and cell transfection

The Mettl3 and Fto cDNA plasmid was constructed by Genechem Co., LTD (Shanghai, China). 2 µg lentivirus was used to transfect neuron with transfection reagents, according to the manufacturers’ instruction for 12 h. At 12 h post-infection, the medium was replaced with the neuron maintenance medium. The gene expression level was analysed by Western blot.

### Western blot analysis

After the neurons were washed with PBS, RIPA lysis buffer (Merck Millipore, Cat. No. 20–188) containing protease inhibitors (Roche, Cat. No. 5,892791,001) was added to fully lyse the cells on ice for 30 minutes, followed by centrifugation at 12,000 X g at 4°C for 10 minutes. The supernatant was collected, and the PierceTM BCA Protein Assay Kit (Thermo Scientific, Cat. No. 23225) was used to determine the protein concentration. Finally, the protein was denatured at 100°C for 10 minutes. 20ug of protein were analysed by immunoblotting using an anti-Mettl3 antibody (Abcam Cat. No. ab195352), an anti-Fto antibody (Abcam Cat. No. ab280081), with an anti-β-actin antibody (Abcam Cat. No. ab8226) used as an internal control.

### Quantitative RT-PCR

Total RNA was extracted from neuron using TRIzol reagent (Invitrogen, Carlsbad, CA, USA) and used as a template to synthesize cDNA with a reverse transcription kit (Takara, Dalian, China). Quantitative reverse transcription PCR (qRT-PCR) was performed using SYBR Green reagents (Takara, Dalian, China), with β-actin used as an internal control. The primers used in the present study are listed in Supplemental Table 2–3

### Data Analysis

Briefly, paired-end reads were obtained from the Illumina HiSeq 4000 sequencer (Illumina, Inc., CA, USA), and the sequencing quality was controlled by filtering for Q30 (an index of sequencing quality that indicates the base sequencing error rate is 1/1000). Adapter sequences and low-quality reads were removed by Cutadapt (v1.9.3., a software programme that finds and removes adapter sequences) [39]. The clean reads from the input libraries were mapped to the reference genome (UCSC MM10) using HISAT2 software [40] (v2.1.0). circRNAs were identified with DCC [41] (v 0.4.7; circRNA detection software) using the HISAT2 alignment results. Subsequently, the clean reads from all libraries were mapped to the reference genome (UCSC MM10) using HISAT2 software (v2.0.4) [40]. Methylated sites on RNAs were identified using Model-based Analysis of ChIP-Seq (MACS) software (v1.4.2) [42]. diffReps (v1.55.6; differential modification site detection software) [43] was used to annotate and identify differentially methylated sites (peaks), and a fold change cut-off >2 and a p-value cut-off <0.05 were set as thresholds. The peaks identified by MACS and diffReps that overlapped with the exons of mRNAs and circRNAs were identified and subjected to further analysis.

The data were presented as means ± standard deviation (SD). The MeRIP qRT-PCR experiments were analysed using GraphPad Prism software (version 7.00; La Jolla, CA, USA); the data analyses for the other experiments were performed using R software (version 3.6.2). One-way analysis of variance (ANOVA) was used for comparisons across multiple groups. Tukey’s post hoc test was used to determine specific between-group differences. Pearson correlation analysis was performed to identify correlations between two variables. A p-value < 0.05 was considered to indicate statistical significance (*, p < 0.05; **, p < 0.01; ***, p < 0.001).

## Results

### m6A modification is abundant on neuronal mRNAs

**Figure 1.**
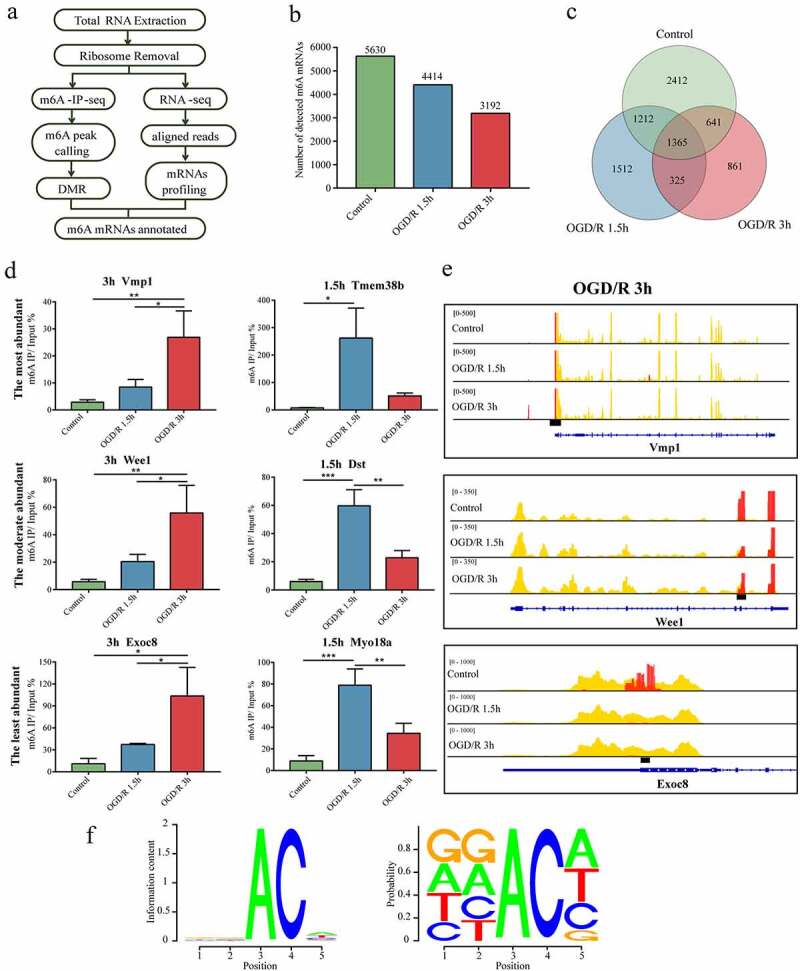
m6A modification is abundant in neuronal mRNAs. (a). Pipeline used to detect m6A mRNAs in neurons. (b). Number of m6A mRNAs in each group. (c). Numbers of OGD/R-specific and common m6A mRNAs. (d). MeRIP qRT-PCR validation of the identified m6A mRNAs. (e). Visualization of the m6A mRNAs selected in Figure E. (f). RRACH conserved sequence motif for the m6A peak regions. *p < 0.05, **p < 0.01, ***p < 0.001.

We first assessed the purity of isolated primary cortical neuron by immunofluorescence, which reached more than 90% (Supplementary Figure 1A). CCK8 assay demonstrated that cell viability declined over time (Supplementary Figure 1B), and LDH release also increased with OGD/R time extension (Supplementary Figure 1C). Those results suggested that our OGD/R model is valid, and could provide a basis for subsequent mechanistic studies. m6A is a well-known internal modification of mRNAs [35,44]. To identify the transcriptome-wide m6A mRNAs of neurons subjected or not to OGD/R, we analysed two OGD/R groups (OGD/R 1.5 h and OGD/R 3 h) and a control group, as shown in the diagram (Figure 1a). To further investigate total m6A modification level in different groups, we conducted m6A dot blot assay. The results indicated that the global m6A level in OGD/R group was reduced (Supplementary Figure 2A). We counted the m6A mRNAs (Figure 1b) and find m6A mRNAs that were shared and specific to each group (Figure 1c). In total, 5630 m6A mRNAs were in control, 4414 in OGD/R 1.5 h, and 3192 in OGD/R 3 h (Figure 1c). Overall, 1365 m6A mRNAs were commonly observed among the three groups (Figure 1c); 2412 m6A mRNAs were specifically observed in the control group, whereas 1512 and 861 were observed in the OGD/R 1.5 h and 3 h groups, respectively.

Based on the fold enrichment of m6A peaks, the m6A mRNAs were divided into three categories as previously reported [25]; the top 5% (most abundant), middle 90% (moderately abundant) and lowest 5% (least abundant). We counted the m6A mRNAs in the three categories in each group and found that most of the m6A mRNAs were moderately modified (Supplementary Figure 2B). Regarding the OGD/R groups, one m6A mRNA was randomly selected from each of the 3 categories for MeRIP qRT-PCR and visualization (OGD/R 1.5 h: Tmem38b (most abundant), Dst (moderately abundant), and Myo18a (least abundant); OGD/R 3 h: Vmp1 (most abundant), Wee1 (moderately abundant), and Exoc8 (least abundant)). The qRT-PCR-determined expression levels of the randomly selected m6A mRNAs were consistent with those of MeRIP sequencing. Visualization of the m6A peaks of the above m6A mRNAs also showed that the abundances of the m6A peaks of the selected m6A mRNAs in the different categories indeed matched the sequencing results (Figures 1E and Supplementary Figure 2C).

m6A modification is easiest to detect in highly conserved regions with a similar sequence: RRACH (R = G or A; H = A, C or U) [45,46]. To clarify whether this consensus sequence existed in the neuronal m6A mRNAs, we analysed all of the m6A peaks of the m6A mRNAs in our data and found that 267,405 peaks had this characteristic sequence, accounting for 61.04% of the total peaks (438,080 peaks). This conserved sequence seems to be a necessity for m6A methylation on neuronal m6A mRNAs (figure 1f).

Next, we identified the m6A mRNAs that were differentially methylated in the OGD/R-treated groups compared with the control group. In total, 292 m6A mRNAs were shared between the groups (subset A), 1622 were unique to the OGD/R 1.5 h group (subset B,) and 206 were unique to the OGD/R 3 h group (subset C) (Supplementary Figure 2C). GO analyses of these three subsets were conducted separately (Supplementary Figure 2D). In each subset, most of the m6A mRNAs were enriched for the cellular process (BP), cell and cell part (CC), and binding (MF) terms (Supplementary Figure 2E). Next, KEGG pathway analyses of the three subsets were conducted (Supplementary Figure 2 F). In subset A, most of the m6A mRNAs were enriched in the cysteine and methionine metabolism and Jak−STAT signalling pathways. In subset B, glycosphingolipid biosynthesis and dopaminergic synapse pathways were enriched. In subset C, the Rap1 signalling, necroptosis and PI3K−Akt signalling pathways were enriched.

### The m6A level does not affect the expression of m6A mRNAs

**Figure 2.**
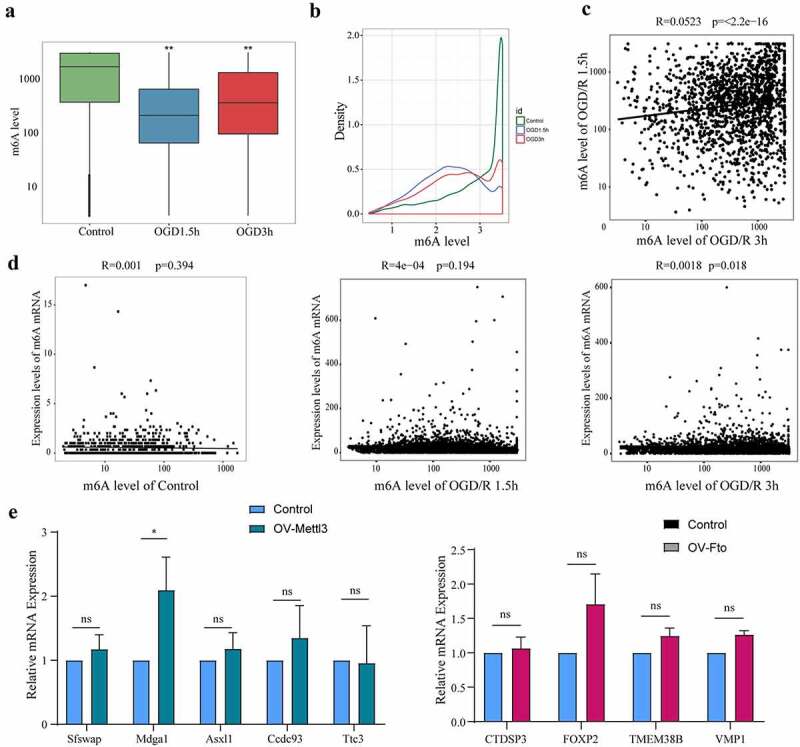
The m6A level does not affect the expression of m6A mRNAs. (a). Comparison of the m6A levels in the mRNAs of different groups. (b). Density distribution of the m6A levels in mRNAs. (c). Linear correlations of the m6A levels among the m6A mRNAs identified in both the OGD/R 1.5 h and OGD/R 3 h groups. (d). Relationships between the expression of mRNAs and the m6A levels in the mRNAs of the control, OGD/R 1.5 h and OGD/R 3 h groups. (e). qRT-PCR showing the expression of the top 5 least abundant-m6A mRNAs after overexpressing Mettl3. (f). qRT-PCR showing the expression of the top 5 most abundant-m6A mRNAs after overexpressing Fto (Hoxd4 could not be detected). *p < 0.05, **p < 0.01, ***p < 0.001, ns: not significant.

In this study, we defined the fold enrichment of m6A peaks in the IP group to input group as the m6A level to assess the degree of m6A modification, as mentioned previously. The m6A level of m6A mRNAs in the OGD/R groups was significantly lower than that in the control group (Figure 2a; p < 0.05), and no difference was found between the OGD/R 1.5 h and OGD/R 3 h groups (Figure 2a). To further understand the levels of m6A modification of the m6A mRNAs in each group, we conducted density analysis. In the control group, most of the m6A mRNAs were highly methylated. In the OGD/R 1.5 h group, most of the m6A mRNAs were highly methylated, but the density was lower than that in the control group. In the OGD/R 3 h group, most of the m6A mRNAs were moderately methylated (Figure 2b). We also analysed the correlations in the m6A level of the m6A mRNAs between the OGD/R 1.5 h group and OGD/R 3 h group and found that they were weakly related, indicating that the change in the m6A level caused by the different OGD/R treatment differed between the two groups (Figure 2c; R = 0.05; p <0.05).

To determine whether m6A mRNA expression was related to the m6A level, another correlation analysis was performed. Unexpectedly, no relationship was found between m6A mRNA expression and the m6A level in the control group (R = 0.004; p >0.05), OGD/R 1.5 h group (R = 4e-4, p >0.05) or OGD/R 3 h group (R = 0.018, p <0.05) (Figure 2d). To verify this conclusion further, we overexpressed Mettl3 (a methylation transferase) (Supplementary Figure 3A) and Fto (a demethylase) (Supplementary Figure 3B) in primary neuron, respectively. In the group overexpressing Mettl3, we selected the top 5 least abundant-m6A mRNAs (Sfswap, Mdga1, Asxl1, Ccdc93 and Ttc3) (Primer sequences used for RT-qPCR have been provided in Table 2) to see whether their expression was affected by Mettl3. The results showed that the expression of the least abundant-m6A mRNAs (except for Mdga1) was not affected by Mettl3 (Figure 2e). In the overexpression Fto group, we also found that the expression of top 5 most abundant-m6A mRNAs (Ctdsp2, Foxp2, Tmem38b, VMP1 and Hoxd4) (except for Hoxd4 that could not be detected) was not affected by Fto (figure 2f). Our findings suggest that the effect of m6A modification on the vast majority of mRNA expression appears to be weak, although there may be a more pronounced effect for individual genes.

**Table 2.** Primer sequences for normal qRT-PCR (mRNA).

Gene	Primer name	Primer sequence
Vmp1	1-Forward	TGGCCTCTCGGATGCTTACA
	1-Reverse	CAGAAGTGTGGAGTGGACTCG
Ttc3	2-Forward	CTTGCCGTTTCTCTCCTTGGA
	2-Reverse	AAACTGGTTTTCGGCTTCAGA
Mdga1	3-Forward	ATGGAGGTGACCTGTCTTCTAC
	3-Reverse	GGCTGGAGCATAGACTCCTT
Asxl1	4-Forward	CTACTCAGATGCTCCAATGACAC
	4-Reverse	TGAAAAGACTAATGCGGCCAG
Sfswap	5-Forward	GAGTACACGGCAGACTCAACT
	5-Reverse	CAGGAAGGGTGCTGTAGTAAGT
VMP1	6-Forward	CCAGAGACGCATAGCAATGAG
	6-Reverse	GCAAGGTAATGAGTGGCTGTC
Tmem38b	7-Forward	TTTTCCCGCACGTCCATGTT
	7-Reverse	GACTCTGCAAGCAGCATACAG
Foxp2	8-Forward	AGTGTGCCCAATGTGGGAG
	8-Reverse	CATGATAGCCTGCCTTATGAGTG
Ctdsp2	9-Forward	AAGGCCCTCTTGTGCTGTTTC
	9-Reverse	GATCGGACTTGGCGATGGT
Hoxd4	10-Forward	AAGTACGTGGACCCCAAGTTT
	10-Reverse	GGGGCACCGTAATGACTTCC

### m6A modification is enriched on neuronal circRNAs

**Figure 3.**
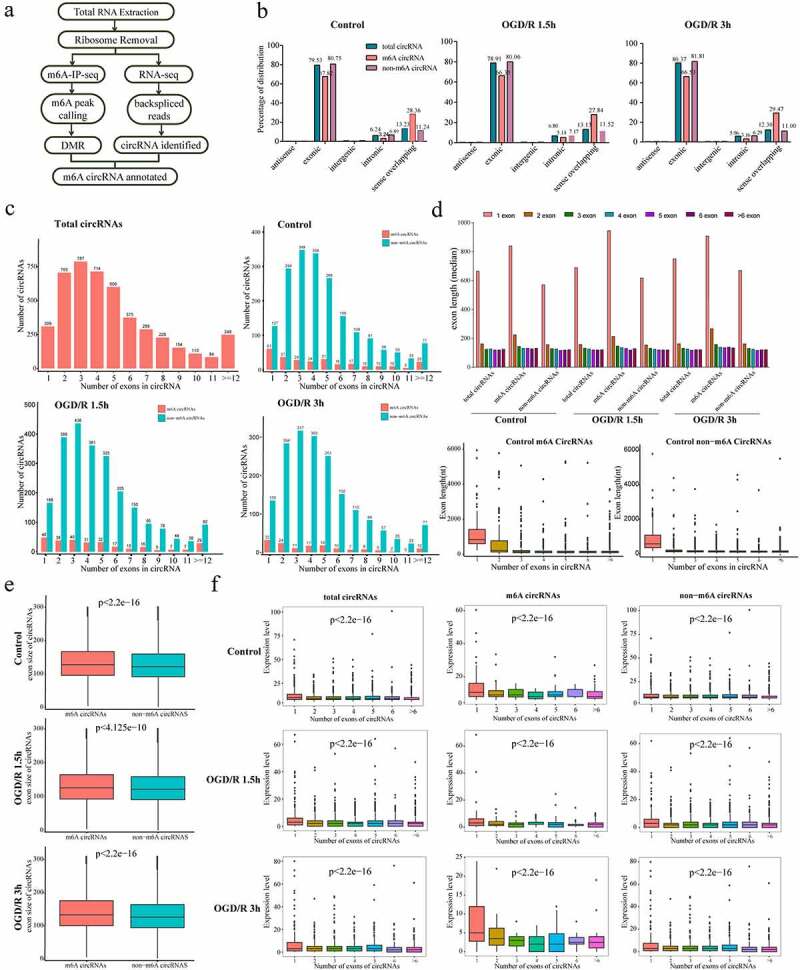
m6A modification is enriched in neuronal circRNAs. (a). Pipeline used to detect m6A circRNAs in neurons. (b). Genomic distributions of total circRNAs, m6A circRNAs and non-m6A circRNAs; the proportions are shown at the top. (c). Numbers of m6A circRNAs and non-m6A circRNAs derived from the different numbers of exons in each group. The number of exons (up to 12) is shown at x axis. (d). Exon lengths of total circRNAs, m6A circRNAs and non-m6A circRNAs. The lengths were calculated based on the numbers of exons spanned by the circRNAs. (e). Comparison of the exon lengths of all m6A circRNAs and non-m6A circRNAs. (f). Expression levels of total circRNAs, m6A circRNAs and non-m6A circRNAs based on the number of exons spanned by each circRNA.

To explore the occurrence of m6A modification on neuronal circRNAs, we constructed libraries for high-throughput sequencing (Figure 3a). The numbers of total circRNAs and m6A circRNAs as well as percentage of m6A circRNAs among the total circRNAs in the different groups were determined by analysis of the raw data (control: 2790 total circRNAs, 864 (30.97%) m6A circRNAs; OGD/R 1.5 h: 3367 total circRNAs, 740 (21.98%) m6A circRNAs; OGD/R 3 h: 2431 total circRNAs, 475 (19.54%) m6A circRNAs)) (Supplementary 4A).

We next analysed the genomic distributions of total circRNAs, m6A circRNAs and non-m6A circRNAs in the control and OGD/R-treated groups. Most of the total circRNAs, m6A circRNAs and non-m6A circRNAs were derived from exonic regions, followed by sense overlapping and intronic regions; a small proportion was derived from intergenic regions (Figure 3b). The resource sequences of the circRNAs were not altered after OGD/R treatment, indicating that OGD/R treatment had little effect on the genomic distributions of the circRNAs. For each genomic source, a small difference between m6A circRNAs and non-m6A circRNAs was found, as the percentage of m6A circRNAs derived from sense overlapping was higher than that of non-m6A circRNAs in each group, suggesting that a small number of circRNAs derived from sense overlapping were more likely to be modified by m6A than other circRNAs (control: 28.36% vs. 11.24%; OGD/R 1.5 h: 27.84% vs. 11.52%; OGD/R 3 h: 29.47% vs. 11.00%)) (Figure 3b).

Because most circRNAs originate from two or three exons [25], we wondered whether the number of exons from which the circRNAs were derived exhibited certain trends and whether the m6A circRNAs had different features. We analysed the number of circRNAs derived from different numbers of exons among the total circRNAs, m6A circRNAs and non-m6A circRNAs in the control and OGD/R-treated groups (Figure 3c). We found that most of the total circRNAs and non-m6A circRNAs were encoded by 3 exons, while most of the m6A circRNAs originated from only 1 exon (Figure 3c). Additionally, the percentages of total circRNAs (m6A circRNAs and non-m6A circRNAs) derived from different numbers of exons did not differ considerably among the control and OGD/R-treated groups, suggesting that the different OGD/R treatments did not affect the number of exons that formed the circRNAs (Figure 3c).

Analysis of the lengths of the exons that generated the circRNAs showed that the exons generating single-exon circRNAs were longer than those generating multiple-exon circRNAs. These findings applied to all the circRNAs in the control and OGD/R-treated groups, which indicated that the different OGD/R treatments did not affect the lengths of exons from which the circRNAs were derived (Figure 3d and Supplementary Figure 4C). We also analysed the median lengths of all exons related to circRNAs (total circRNAs, m6A circRNAs, and non-m6A circRNAs) in the control, OGD/R 1.5 h and OGD/R 3 h groups. The median lengths of all exons generating m6A circRNAs were greater than those generating non-m6A circRNAs in all three groups (Figure 3e).

We next investigated whether the number of exons from which each circRNA was derived could influence the expression of circRNAs. Single-exon circRNAs were more abundant than multiple-exon circRNAs among the total, m6A and non-m6A circRNAs in the three groups (p <2.2e−16) (figure 3f).

The m6A modification on mRNAs has a conserved sequence (RRACH (R = G or A; H = A, C or U)); thus, we wondered whether sequence preference existed in the m6A circRNAs. In total, 35,955 peaks (60.00%) had this characteristic among 59,917 peaks. This conserved sequence seemed necessary for the m6A methylation of neuronal m6A circRNAs (Supplementary Figure 4B).

### The m6A level does not affect the expression of m6A circRNAs

**Figure 4.**
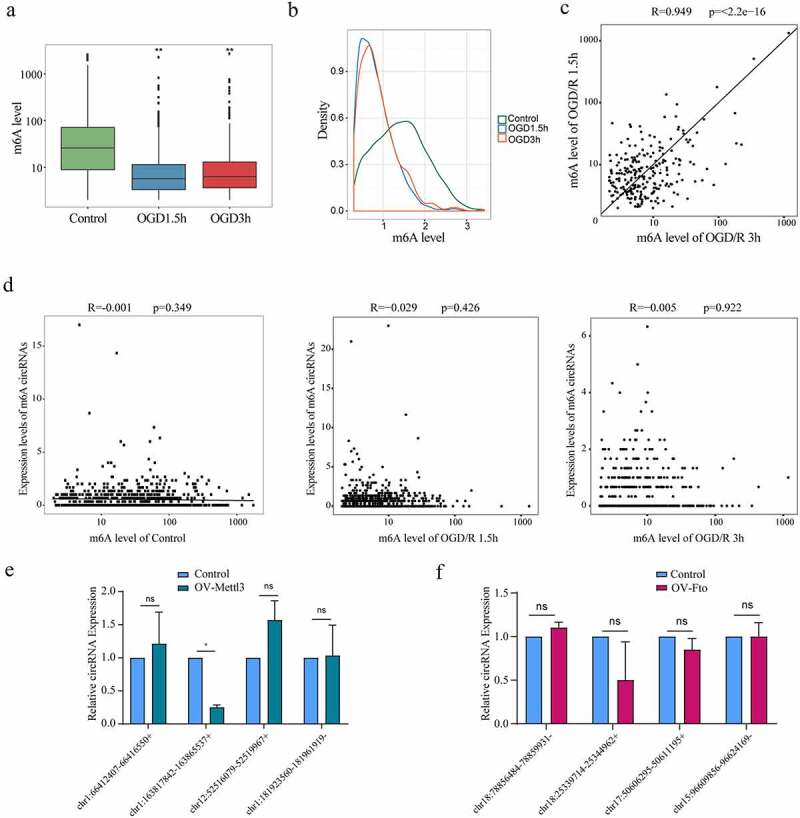
The m6A level does not affect the expression of m6A circRNAs. (a). Comparison of the m6A levels in the circRNAs in different groups. (b). Density distribution of the m6A levels in circRNAs. (c). Linear correlations of the m6A levels among the m6A circRNAs identified in both the OGD/R 1.5 h and OGD/R 3 h groups. (d). Relationship between the expression of circRNAs and the m6A levels in the circRNAs of the control, OGD/R 1.5 h and OGD/R 3 h groups. (e). qRT-PCR showing the expression of the top 5 least abundant-m6A circRNAs after overexpressing Mettl3 (chr16:94403314-94468060+ could not be detected). (f). qRT-PCR showing the expression of the top 5 most abundant-m6A circRNAs after overexpressing Fto (chr11:5329879-5338763- could not be detected). *p < 0.05, **p < 0.01, ***p < 0.001, ns: not significant.

The degree of m6A modification of m6A circRNAs in neurons remains unknown. We evaluated the m6A levels of m6A circRNAs in the control and OGD/R-treated groups and found that the levels in the OGD/R-treated groups were significantly lower than that in the control group (both p <0.05) but did not differ between the OGD/R-treated groups (Figure 4a). This finding indicated that the OGD/R treatments reduced the m6A level of m6A circRNAs but that the duration of OGD/R treatment did not differentially affect the m6A levels. This result is similar to that for the m6A levels of the m6A mRNAs. To further understand the m6A circRNA and m6A levels in each group, we conducted density analysis. In the control group, most m6A circRNAs were highly methylated. In the OGD/R-treated groups, most m6A circRNAs were poorly methylated (Figure 4b). This result partly explains why the m6A levels of m6A circRNAs were low in the OGD/R-treated groups. We also investigated whether the m6A level of m6A circRNAs in the OGD/R-treated groups were related through correlation analysis. The results showed that the m6A levels in the two groups were positively correlated (Figure 4c; R = 0.9, p <0.05), indicating that the changes in m6A circRNA m6A levels caused by the different OGD/R treatments were consistent between groups.

To determine whether m6A circRNA expression was related to the m6A level, another correlation analysis was performed. Surprisingly, no relationship was found between m6A circRNA expression and the m6A level in the control (R = 0.001, p >0.05), OGD/R 1.5 h (R = −0.029, p >0.05) or OGD/R 3 h groups (R = −0.005, P >0.05) (Figure 4d), and the results differed from those reported in a previous study [25], in which m6A circRNA expression was positively correlated with the m6A levels in HeLa cells. We also performed experimental validation in neurons overexpressing Mettl3 and Fto by detect the expression of corresponding m6A circRNAs. In the group overexpressing Mettl3, we selected the top 5 least abundant-m6A circRNAs (chr16:94403314-94468060+, chr1:163817842-163865537+, chr1:181923560-181961919-, chr12:52516079-52519967+, chr1:66412407-66416550+) (Primer sequences used for RT-qPCR have been provided in Table 3) to see whether their expression was affected by Mettl3. The results showed that most of the least abundant-m6A circRNAs’ expression was not affected by Mettl3 (chr16:94403314-94468060+ could not be detected). However, after overexpression of Mettl3, the expression level of chr1:163817842-163865537+ was significantly reduced (Figure 4e). In the overexpression Fto group, we also found that the expression of top 5 most abundant-m6A circRNAs (chr18:25339714-25344962+, chr18:78856484-78859931-, chr17:50606295-50611195+, chr15:96609856-96624169-, chr11:5329879-5338763-) (figure 4f) was not affected by Fto except for the chr11:5329879-5338763- (could not be detected). The above results indicate that the majority of circRNA expression is not affected by m6A modifications.

**Table 3.** Primer sequences for normal qRT-PCR (circRNA).

Gene	Primer name	Primer sequence
chr18:25339714-25344962+	1-Forward	GAGTGTCTCTGGACCAAGATAACC
	1-Reverse	TTTTGTGGTCTGGCTACACTCA
chr18:78856484-78859931-	2-Forward	TCCTCCATGTCTCCAGGCATAC
	2-Reverse	CCATTCCATGTGGCTCTGACT
chr17:50606295–50611195+	3-Forward	GTAACAACTTATGACATGGATGGCA
	3-Reverse	CCATTGAGTTGATACAGTCACTTGC
chr15:96609856-96624169-	4-Forward	TCTTTAACCTGAGCAACGCC
	4-Reverse	AATACAGATGCCTGAGACCCAGA
chr11:5329879-5338763-	5-Forward	ACAGTTGAACTGCAGAGACAGATG
	5-Reverse	GGCTGTTCAAGCTTCACCACT
chr16:94403314-94468060+	6-Forward	GATTCCCAGAAACCAAATGCA
	6-Reverse	CCCACACACTGATTCTCACACAA
chr1:163817842-163865537+	7-Forward	GATGTCATAATCAAGGAAACACACC
	7-Reverse	CCGCAAGATTCAAAAGCAAGTA
chr1:181923560-181961919-	8-Forward	AGAATGTCCAATGCTGTGAACA
	8-Reverse	CTCTGAATGTGTTGTTGCCTGTA
chr12:52516079-52519967+	9-Forward	GGCAGAATTTCCACTCCTGAAAG
	9-Reverse	CATCTTCGAGGCTGTAAGTAAATCTT
chr1:66412407-66416550+	10-Forward	ACAGTTGAACTGCAGAGACAGATG
	10-Reverse	GGCTGTTCAAGCTTCACCACT

### Crosstalk between m6A mRNAs and m6A circRNAs

**Figure 5.**
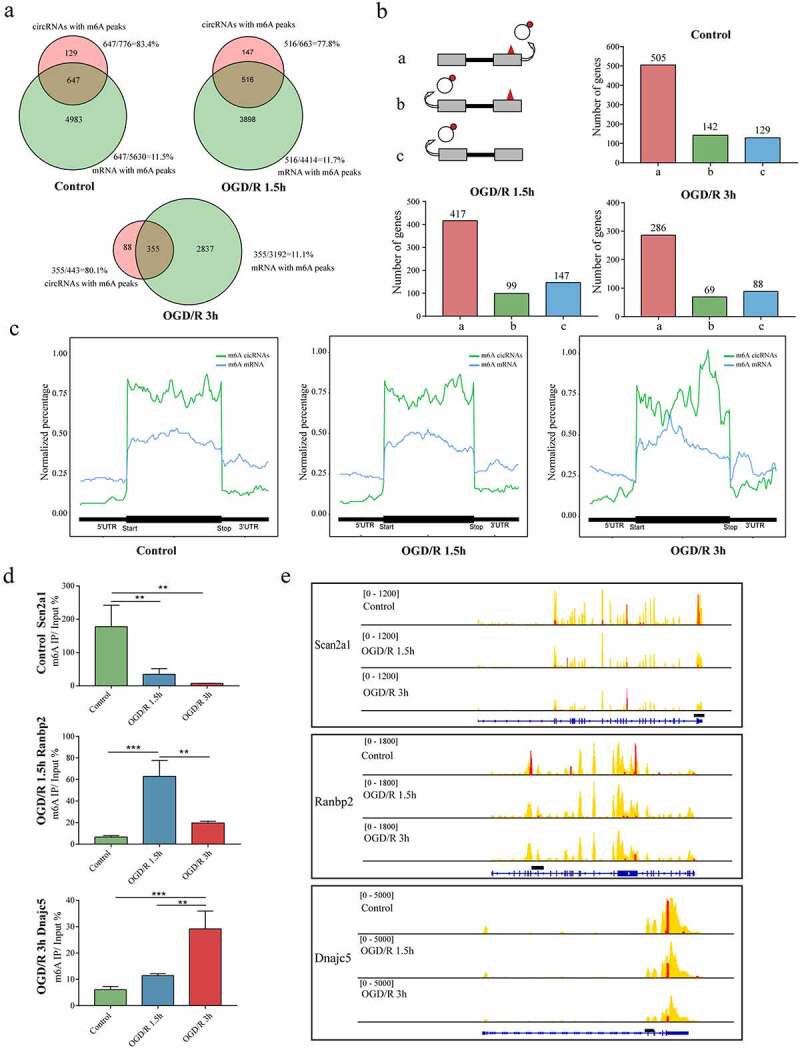
Crosstalk between m6A mRNAs and m6A circRNAs. (a). Venn diagram showing the overlapping genes encoding m6A circRNAs and m6A mRNAs. The total number and percentage for each part are shown. (b). m6A modifications in mRNAs and circRNAs. In the first pattern, m6A circRNAs were produced from exons with m6A modifications, and m6A mRNAs were produced from the same exons (a). In the second pattern, m6A circRNAs were produced from exons differing from those encoding m6A mRNAs (b). In the third pattern, m6A circRNAs were produced from exons that did not produce m6A mRNAs (c). **(C)**. Distributions of exons encoding circRNAs and m6A circRNAs across genes compared with the m6A peak distributions on linear RNAs. (d). MeRIP qRT-PCR validation of the identified genes containing m6A peaks that could generate m6A mRNAs and m6A circRNAs. (e). Visualization of genes containing m6A peaks that could generate m6A mRNAs and m6A circRNAs.

Both m6A mRNAs and m6A circRNAs are derived from precursor mRNAs (pre-mRNAs) [44,47]. We investigated whether m6A circRNAs and m6A mRNAs could be derived from the same genes and found that in the control group, 83.37% of the parental genes of m6A circRNAs also encoded m6A mRNAs; this subset of genes accounted for only 11.49% of the parental genes of m6A mRNAs. The two percentages in the OGD/R-treated groups were similar to those in the control group (Figure 5a).

Next, we sought to determine whether certain production patterns govern m6A circRNA biogenesis. Three production patterns of m6A circRNAs were identified: a. m6A circRNAs were produced from m6A exons that also encoded m6A mRNAs; b. m6A circRNAs were produced from regions of genes other than m6A exons that could encode m6A mRNAs; c. m6A circRNAs were produced from genes that could not encode m6A mRNAs (Figure 5b). In pattern a, 505 genes (65.07%) in the control and 417 (62.89%) and 286 (64.56%) genes in the OGD/R 1.5 h and 3 h groups, respectively, were observed. In pattern b, 142 (18.30%) genes in the control and 99 (14.93%) and 69 (15.57%) genes in the OGD/R 1.5 h and 3 h groups, respectively, were observed. In pattern c, 129 genes (16.62%) in the control and 147 (22.17%) and 88 genes (19.86%) in the OGD/R 1.5 h and 3 h groups, respectively, were observed. These results indicated that the three different production patterns participated in the production of m6A circRNAs and different OGD/R treatments did not affect the proportions of m6A circRNAs produced from these three patterns.

We analysed the m6A peak distribution among m6A mRNAs and m6A circRNAs along a metagene (Figure 5c). For each group (control, OGD/R 1.5 h, and OGD/R 3 h), we found that the m6A modifications were mostly concentrated in the CDS region of the mRNAs and at the end of the CDS region near the 3ʹUTR in circRNAs.

To confirm the existence of m6A modifications on transcripts that could encode both m6A mRNAs and m6A circRNAs, we selected one specifically expressed gene from each group (control: Scan2a1; OGD/R 1.5 h: Ranbp2; OGD/R 3 h: Dnajc5) for visualization (Figure 5d). Subsequently, we designed primers to perform MeRIP qRT-PCR (Figure 5e) and found that the m6A circRNAs selected from the different groups were indeed specifically expressed.

### m6A circRNAs show time-specific methylation patterns under different OGD/R treatments

**Figure 6.**
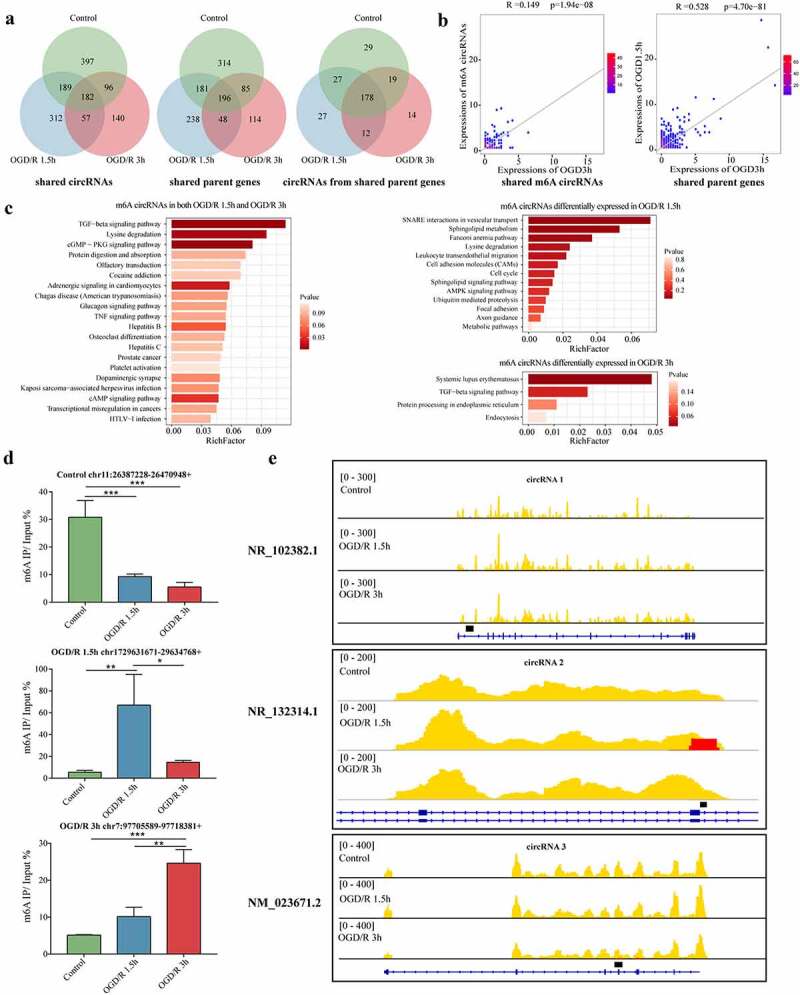
m6A circRNAs have time-specific methylation patterns with different OGD/R treatments. (a). Venn diagram showing the shared circRNAs, shared circRNA parent genes and circRNAs derived from those shared genes in the different OGD/R groups. The numbers of circRNAs are shown. (b). Two-dimensional histograms comparing the expression levels of the m6A circRNAs in the OGD/R 1.5 h and OGD/R 3 h groups. The colour represents the number of circRNAs expressed in both the OGD/R 1.5 h and OGD/R 3 h groups. (c). KEGG pathway analysis of the shared and specifically expressed m6A circRNAs in the OGD/R 1.5 h and OGD/R 3 h groups. (d). MeRIP qRT-PCR validation of m6A circRNAs specifically expressed in the control, OGD/R 1.5 h and OGD/R 3 h groups. (e). Visualization of specifically expressed m6A circRNAs in the control, OGD/R 1.5 h and OGD/R 3 h groups.

Different OGD/R treatments can cause changes in mRNA metabolism [48]. In this study, we were interested in whether different durations of OGD/R treatment could affect the metabolism of m6A circRNAs. We found that 182 m6A circRNAs were shared among the control and OGD/R-treated groups; 397 existed in only the control, whereas 312 and 140 existed in the OGD/R 1.5 h and 3 h groups, respectively (Figure 6a, left). We next analysed the parental genes of the m6A circRNAs in each group. In total, 196 parental genes were shared; 314 parental genes were specific to the control group, whereas 238 and 114 were specific to the OGD/R 1.5 h and 3 h groups (Figure 6a, centre). Next, we analysed m6A circRNAs derived from the shared parental genes (196). These genes produced different m6A circRNAs under the different OGD/R treatments (Figure 6a, right). Then, we conducted correlation analyses of the expression of shared m6A circRNAs and shared parent genes. We found little correlation in the expression of shared m6A circRNAs between in the OGD/R-treated groups (R = 0.12, p <0.001) (Figure 6b, left), but the expression of the shared parent genes between the OGD/R-treated groups was moderately correlated (R = 0.53; p <0.001) (Figure 6b, right).

Next, we identified differentially methylated m6A circRNAs in the OGD/R-treated groups compared with the control group. The shared and unique differentially methylated m6A circRNAs in the OGD/R-treated groups were analysed (Supplementary Figure 5A). GO analysis was conducted on the differentially methylated m6A circRNAs shared between the OGD/R-treated groups (subset 1) and on the differentially methylated m6A circRNAs unique in the OGD/R 1.5 h (subset 2) and OGD/R 3 h (subset 3) groups (Supplementary Figure 5B). KEGG pathway analysis was also employed to identify the enriched signalling pathways among the three subsets. In subset 1, most of the m6A circRNAs were involved in the TGF-beta signalling pathway and cGMP−PKG signalling pathway, which are both related to apoptosis and the cell cycle [49,50]. In subset 2, SNARE interactions in vesicular transport, sphingolipid metabolism, the AMPK signalling pathway and axon guidance were the main enriched pathways; the AMPK signalling pathway also plays an important role in apoptosis [51]. In subset 3, the systemic lupus erythematosus and TGF-beta signalling pathways were the main enriched pathways (Figure 6c).

To determine the actual m6A level of the m6A circRNAs, three specifically expressed m6A circRNAs were selected (control: chr11:26387228-26470948+ (circRNA 1), OGD/R 1.5 h: chr17:29631671-29634768+ (circRNA 2), OGD/R 3 h: chr7:97705589-97718381+ (circRNA 3)), to perform MeRIP qRT-PCR. The m6A level of circRNA 1 was significantly higher in the control group (Figure 6d; both p <0.05); similarly, the m6A level of circRNA 2 was higher in the OGD/R 1.5 h group than in the other two groups (both p <0.05), and the m6A level of circRNA 3 was higher in the OGD/R 3 h group than in the other two groups (both p <0.05) (Figure 6d). The m6A modification peaks of the selected m6A circRNAs were then intuitively analysed through visualization (Figure 6e), and the results were almost identical to the MeRIP qRT-PCR results. This finding suggested that the m6A levels of the m6A circRNAs were specific to the different groups.

## Discussion

m6A is one of the most abundant modifications among all eukaryotes [52,53], as approximately 0.4–0.6% of all cellular RNAs have this modification [44,52]. Additionally, m6A accounts for more than 80% of all RNA base methylations and exists in various species [54–56]. Due to technical limitations, most previous studies on m6A modification have focused on mRNAs. m6A modification of mRNAs affects different aspects of mRNA metabolism, including splicing [48], translation [57,58], degradation [15] and phase separation potential [59]. m6A modification of mRNAs also plays important roles in many diseases, such as glioblastoma [60], acute myeloid leukaemia [61] and hepatocellular carcinoma [62]. In recent years, with the improvements of RNA sequencing and computational approaches, increased attention has been given to the effects of m6A modifications on non-coding RNAs, particularly circRNAs [28,63]. The number of studies on m6A circRNAs is increasing, but few studies have been reported on m6A circRNAs in the context of diseases and pathological processes. The characteristics of m6A circRNAs in normal OGD/R-treated neurons remain unclear [45].

Gradually, studies have focused on the linkage between mRNAs and circRNAs of the same genetic origin. Liu et.al found that the expression of circIGF2BP3 significantly correlated with METTL3 expression and m6A recognition can influence the ratio of IGF2BP3-generated mRNA to circRNA [31]. Our results also suggest that there may be a crosstalk between m6A mRNA and circRNA in neurons. In terms of the proportion of m6A mRNA and circRNA, the different OGD/R treatments seem to have no effect on the proportion of m6A mRNA and circRNA produced. From the available studies, circRNA and mRNA derived from the same genetic origin may have same and different functions. Yang et.al report circ-FBXW7 and a functional protein encoded by the circRNA, which could inhibit proliferation and cell cycle acceleration in tumour [64]. The function of this circRNA is similar to the function of the corresponding mRNA. SHPRH is another gene, which could encode both mRNA and circRNA. mRNA of SHPRH has multiple functions, such as DNA repair [65], rRNA transcription regulation [66]. Circular SHPRH could encode a 17 kDa protein and suppresses glioma tumorigenesis. We speculate that the production of m6A mRNA or circRNA for the same gene may be important for physiological or pathological state regulation. We are also curious about what exactly determines the generation of this m6A mRNA or circRNA preference to. Currently, we hypothesize that the generation of this preference may be related to the recognition of m6A, and that the differential expression of a certain recognition protein in some cases may lead to the generation of different kinds of RNAs and thus affect different functions, however, this requires further bioinformatics analysis and experimental validation. In addition, based on existing studies, we speculate that there may also be phase separation-related patterns that play a role in the production of m6A mRNA and circRNA. In this study, we identified three methylation patterns of m6A circRNAs. A possible mechanism of m6A’s roles is depicted as follows: m6A circRNAs and m6A mRNAs encoded by the same exons may be bundled as part of a chromatin-associated liquid phase transition, leading to the formation of nuclear ‘liquid droplets’ that are critical for maintaining the structure and function of the nucleolus [67,68]. In contrast, m6A circRNAs arising from non-methylated regions of genes may not be integrated with m6A mRNAs. Different elements (combined m6A circRNAs and m6A mRNAs, separate m6A circRNAs) may carry different information to assist in the process of nucleolar liquid-liquid phase separation. These different information-carrying elements might also be transmitted to the cytosol from the nucleus and participate in the production of other mRNAs [69,70]. However, further experimental studies are needed to fully confirm the hypothesis mentioned above.

Some studies have shown that m6A modification is cell-type-specific [25,71]. Previous studies have suggested the presence of specific m6A mRNAs in embryonic stem cells (ESCs), induced pluripotent stem cells (iPSCs), neural stem cells (NSCs), and testicular Sertoli cells (SCs). Additionally, the m6A modifications in different regions of mRNAs are variable in different cells. Our results suggest that most m6A mRNAs have m6A modifications in the central CDS region and that most m6A circRNAs have m6A modifications at the end of the CDS region near the 3ʹUTR. Most of the identified m6A peaks contained a conserved RRACH motif, but a few other motifs existed with cell-type specificity [71]. We observed the RRACH motif relatively uniformly, and it might be a characteristic conserved sequence modified by m6A in neurons. The view that m6A modification is cell-type specific is mostly derived from studies on m6A mRNAs; few researchers have investigated the m6A modification of circRNAs in different cell types. Zhou et al. [25] studied the characteristics of m6A circRNAs in human ESCs (hESCs). We showed that the total number of m6A circRNAs in neurons was less than that in hESCs and that the distribution of m6A was different between neurons (CDS region near the 3ʹUTR) and hESCs (3ʹUTR). The difference in the modified region may affect the regulatory mechanisms of m6A circRNAs (such as their miRNA-sponging functions) and may be a manifestation of the neuronal specificity of m6A modification. The m6A modification levels on RNAs can affect the expression of these RNAs in some cell lines [25]. Our results suggest that the m6A modification level does not affect the expression level of either mRNAs or circRNAs in primary neurons. This might also be a cell type-specific characteristic of primary neurons. We are also interested in why the cell-type-specificity exist. Based on previous studies, we found that the expression of m6A regulators in different cell states or diseases seems to be inconsistent [72,73]. Thus, we speculate that the expression pattern of overall m6A molecules in different cell types may be a possible reason for the cell type specificity of m6A modifications in different cells. Studying the expression patterns of multiple m6A regulators in different cell types may be an important aspect in elucidating the cell type specificity of m6A.

M6A modifications are time-specific in developmental processes as well as in some diseases [74–77]. Lence et al. reported that there are dynamic changes in m6A level at different stages of Drosophila development [75]. In the developing mouse cerebellum, Ma et.al uncovered distinct features of temporal-specific m6A methylation across the four postnatal developmental processes [74]. In Alzheimer’s disease, m6A level also displays temporal and spatial dynamics during neurodevelopment and ageing [76]. Mathiyalagan et.al reported that the significantly elevated levels of m6A in RNA extracted from human, pig, and mouse failing left ventricular explants and a sustained increase in m6A RNA in chronic phases of MI-induced heart failure [77]. In the present study, we focused on the changes of m6A circRNA under different OGD/R treatments. This change characteristic is similar to the above studies, indicating that m6A circRNAs in neurons is also time-specific after being subjected to different time OGD/R treatments. The production of different m6A circRNAs at different OGD/R treatments may affect multiple functions, as circRNAs themselves regulate multiple biological processes through ceRNA machinery, transcriptional regulators, and other roles. This time-specific production may correspond to multiple physio pathological changes that affect the recovery of cells after injury. For time-specific regulation, we still believe that changes in m6A regulator may be the main reason, and of course further studies need to be carried out.

## Conclusions

Our findings highlight the m6A modification profiles of the mRNAs and circRNAs in neurons and neurons under different OGD/R conditions. We also identified crosstalk between m6A mRNAs and m6A circRNAs. Distinct OGD/R treatments induced the generation of different m6A circRNAs from the same genes. These results provide epigenetic evidence of potential treatments for diseases related to the pathological process of OGD/R.

## Supplementary Material

Supplemental Material
