# Supplementary material for: Crosstalk between m6A mRNAs and m6A circRNAs and the time-specific biogenesis of m6A circRNAs after OGD/R in primary neurons
Source: Epigenetics. 2023 Mar 1;18(1):2181575. doi: 10.1080/15592294.2023.2181575 (PMC9988353; doi:10.1080/15592294.2023.2181575)
Supplement: Supplemental Material [file KEPI_A_2181575_SM3102.zip › Supplementary files/Supplementary Figure Captions.docx]

**Supplementary Figure Captions**

**Supplementary Figure 1.** Preparation of OGDR model. **(A)**. Identification of primary mouse cortical neurons. Red: β-III tubulin; green: GFAP; blue: DAPI, Scale bar: 50 µm. The purity of neurons in 3 independent repeated experiments were quantified. **(B)**. Cell viability was assessed after different OGD/R duration using the CCK8 colorimetric assay. **(C)**. LDH release assay detects impairment of neurons at different OGD/R times. *p<0.05, **p<0.01, ***p<0.001

**Supplementary Figure 2.** m6A modification is abundant on neuronal mRNAs. **(A)**. m6A antibody dot blot staining of total RNA (300 ng left and 150ng right) from different OGD/R neuron lysate. **(B)**. The numbers of mRNAs in different modification categories. **(C)**. Visualization of the selected m6A mRNAs. **(D)**. Venn diagram showing the differentially methylated m6A mRNAs in OGD/R 1.5 h group and OGD/R 3 h group. **(E)**. GO analysis of three subsets of differentially methylated m6A mRNAs. **(F)**. KEGG pathway analysis of three subsets of differentially methylated m6A mRNAs, the relevant pathways are highlighted in red.

**Supplementary Figure 3.** Overexpression of Mettl3 and Fto in primary neuron. **(A-B)**. Western blot analysis showing the protein expression of Mettl3 and Fto after overexpression lentivirus transfection. The expression levels were quantified and normalized to that of β-actin. *p<0.05, **p<0.01.

**Supplementary Figure 4.** m6A modification is enriched on neuronal circRNAs. **(A).** The numbers of total circRNAs and m6A circRNAs in different groups. **(B)**. The conserved motif analysis of m6A circRNAs. **(C)**. Exon lengths of total circRNAs, m6A circRNAs and non-m6A circRNAs in OGD/R 1.5 h group and OGD/R 3 h group.

**Supplementary Figure 5.** m6A circRNAs had time-specific methylation patterns under different OGD/R treatments. **(A)**. Venn diagram showing the differentially methylated m6A circRNAs in OGD/R 1.5 h group and OGD/R 3 h group. **(B)**. GO analysis of three subsets of differentially methylated m6A circRNAs.
